# SSF-DDI: a deep learning method utilizing drug sequence and substructure features for drug–drug interaction prediction

**DOI:** 10.1186/s12859-024-05654-4

**Published:** 2024-01-23

**Authors:** Jing Zhu, Chao Che, Hao Jiang, Jian Xu, Jiajun Yin, Zhaoqian Zhong

**Affiliations:** 1 Key Laboratory of Advanced Design and Intelligent Computing, Ministry of Education, Dalian University, Dalian, 116000 China; 2 School of Software Engineering, Dalian University, Dalian, 116000 China; 3 General Surgery, Affiliated Zhongshan Hospital of Dalian University, Dalian, 116000 China

**Keywords:** DDI prediction, Molecular graph, Sequence feature, Substructure interactions, Deep learning

## Abstract

****Background**:**

Drug–drug interactions (DDI) are prevalent in combination therapy, necessitating the importance of identifying and predicting potential DDI. While various artificial intelligence methods can predict and identify potential DDI, they often overlook the sequence information of drug molecules and fail to comprehensively consider the contribution of molecular substructures to DDI.

****Results**:**

In this paper, we proposed a novel model for DDI prediction based on sequence and substructure features (SSF-DDI) to address these issues. Our model integrates drug sequence features and structural features from the drug molecule graph, providing enhanced information for DDI prediction and enabling a more comprehensive and accurate representation of drug molecules.

****Conclusion**:**

The results of experiments and case studies have demonstrated that SSF-DDI significantly outperforms state-of-the-art DDI prediction models across multiple real datasets and settings. SSF-DDI performs better in predicting DDI involving unknown drugs, resulting in a 5.67% improvement in accuracy compared to state-of-the-art methods.

## Introduction

In the clinical field, multi-drug combination therapy has become increasingly popular because this therapeutic approach is known to enhance the treatment efficacy and provide a broader range of treatment options. However, the complex nature of multiple drug interactions combined with individual variability can increase the likelihood of adverse side effects [1]. Adverse drug reaction events in the United States alone incur an annual expenditure exceeding $10 billion, with over 30% attributed to drug–drug interactions (DDI) [2]. Affected by the recent COVID-19 pandemic, many infected patients with pre-existing conditions, such as cardiovascular disease or diabetes, must take antipyretics and treatments for COVID-19 alongside their regular medications [3], increasing their risk of clinical side effects. Since traditional drug screening methods are expensive and time-consuming [4], developing more efficient and accurate drug interaction prediction methods is crucial to guide drug development.

Existing computational methods for drug–drug interaction prediction methods can be divided into two categories: traditional machine-learning methods and deep learning-based methods. Various traditional machine learning methods rely on the drug similarity assumption [5], where it is believed that if drugs A and B interact to produce a specific biological effect, then drugs similar to one of drugs A and B are likely to interact with the other one to produce the same effect. Therefore, Drugs are processed depending on their similarities in chemical structures, individual side effects, targets, and pathways. However, the features for which they show some similarity might be irrelevant to the prediction task of concern [6]. Furthermore, these methods often rely on hand-crafted features and domain knowledge support [7], which makes these methods unsuitable for application in the development phase [8].

A significant increase has been recorded in drug data, along with the increasing computational power of hardware devices in recent years, paving the way for the widespread use of deep learning in drug interaction prediction [9], showing more encouraging performance than traditional machine learning methods [10]. Current deep learning-based DDI prediction methods are roughly divided into two categories: methods that rely on the prediction of drug molecular sequence features and those that rely on the structure of the drug molecular graph. The former category involves processing the drug SMILES sequence. SMILES [11] is a string representing the structure of a chemical molecule, which can transform complex drug molecular structures into a form that a computer can process. SPE [12] enhances atom-level tokenization by labeling and training SMILES sequences, making them useful for molecule generation and other tasks. MCANet [13] extracts features of drug sequence and protein sequence using a cross-attention mechanism for drug–target interaction prediction, resulting in high prediction accuracy. However, relying solely on SMILES sequence features is limited in capturing molecules’ two- or three-dimensional spatial structure information and disregard important topological features.

Graph Neural Networks (GNNs) have demonstrated remarkable abilities in characterizing and learning the complex structures of drug molecules [14], which has led to the development of methods for prediction through drug graph structures. Existing DDI prediction methods based on GNNs typically leverage the topological and semantic modeling capabilities of GNNs to represent drugs. Then, they learn the representations of drug pairs by considering the respective representations of each drug involved [15]. MR-GNN [16] uses an end-to-end GNN to obtain graph-structured entity structural features. Molormer [17] uses the two-dimensional structural information of a drug as input and encodes the molecular graph with spatial information for DDI task prediction. A drug can also be divided into several functional groups or chemical substructures, leading to certain pharmacological properties [18]. Some studies have predicted DDI based on information about drug molecule substructures, such as GMPNN-CS [19], DGNN-DDI [20], DDI-SSL[21] and SSI-DDI [22]. DDI is a complex reaction process encompassing knowledge from multiple domains, including biology and chemistry. Prediction methods based on molecular structures primarily emphasize the topology of atoms and bonds within drug molecules. However, if two drugs have similar molecular structures but different sequences, these models cannot distinguish their subtle differences well. In such scenarios, predicting drug interactions relies solely on the molecular map structure is challenging. Consequently, predicting DDI solely based on drug molecular diagram structures may lead to insufficient accuracy. There are also innovative methods that utilize multimodal data or drug interaction information for prediction, yielding superior results, DPSP[23] predicts DDIs using a multimodal framework through drug substructure information as well as mono side effects, target proteins, enzymes, and pathways. NNPS [24] Predicts polypharmacy side effects by using novel feature vectors based on mono side effects, and drug–protein interaction information.

Overall, numerous models have been developed for the prediction of DDI, demonstrating promising performance. Nonetheless, deep learning-based DDI prediction methods often fail to adequately address at least three prominent issues. Firstly, relying solely on drug molecular graph structures or sequence features provides limited drug embedding information, restricting the DDI prediction performance. Secondly, some methods based on the structure of drug molecular maps are designed to capture the entire molecular structure of drugs for prediction, while it has been proven that DDIs mainly depend on only a subset of the whole chemical structure [22]. Considering the entire molecular structure for DDI prediction may introduce bias by incorporating irrelevant data, often leading to the oversight of crucial drug molecule substructural information [6]. Thirdly, Most research primarily focuses on conducting transductive experiments involving training and test sets of public drugs. However, in real-world scenarios, experiments in the inductive setting are often necessary to infer interactions between newly introduced drugs and existing drugs.

In this paper, we propose a novel DDI prediction deep learning model for DDI prediction based on sequence and substructure features (SSF-DDI) to overcome the abovementioned limitations. Firstly, we used a Convolutional Neural Network (CNN) to extract drug sequence features, and the Mix-attention mechanism was used to determine the importance weight by learning the interaction scores between the sequence features of two drugs. Second, we constructed a novel drug substructure graph feature encoder called SGFE to extract drug substructure features. After that, SSF-DDI combines sequence and substructure features and leverages the fusion of these features to predict drug interactions. The research contributions are summarized as follows:

a. We proposed a novel DDI prediction model, SSF-DDI, that combines drug sequence features and drug molecule graph structural features. Our model captures a broader range of feature information for DDI prediction by incorporating the topological characteristics of drug molecules and sequence features. This comprehensive approach enables a more accurate and comprehensive representation of drug molecule features.

b. We introduced a novel drug substructure graph feature encoder (SGFE), which effectively extracts drug atoms and drug molecule structural features.

c. We performed experiments on both transductive and inductive settings, demonstrating that our model outperforms other approaches. Comprehensive experimental evaluations on DrugBank and Twosides indicate that SSF-DDI achieves an accuracy of 96.45% in the transductive setting, with a relative accuracy improvement of 0.36%. In the inductive environment, SSF-DDI achieves an accuracy of 87.3% in predicting new drugs, with an accuracy improvement of 5.67%. Furthermore, to explore its applicability in real-world scenarios, we conducted experiments in an inductive environment, where SSF-DDI exhibited effectiveness in predicting drug–drug interactions (DDI) for newly approved drugs and demonstrated transferability in predicting drug combinations. Additionally, to investigate the impact of various components on SSF-DDI, we conducted ablation experiments, revealing that the integrated use of drug sequence features and structural features significantly enhances performance.

Drawing from the literature [25], the utilization of computer-aided drug design has become a prevalent approach in the development of new drugs, as evidenced by the discovery of anti-colorectal cancer drugs targeting wild-type and mutant p53 [26]. Notably, drugs such as imatinib (Gleevec), employed in the treatment of chronic myeloid leukemia (CML) [27], highlight the widespread adoption of computer-aided design in current drug development practices. Therefore, our approach holds promising prospects. Collectively, these results indicate that SSF-DDI serves as a robust tool for DDI prediction and holds practical significance in real-world applications, playing a promising role in drug design and discovery research.

## Method

**Fig. 1:**
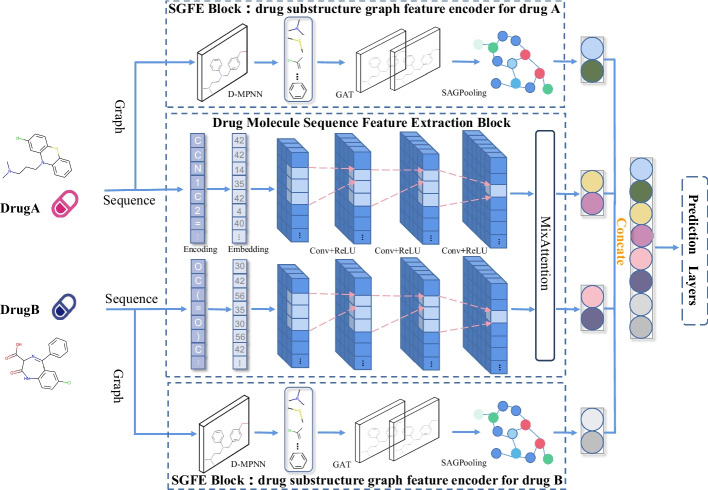
Overview of SSF-DDI method. SSF-DDI sequence feature encoder captures sequence features are extracted from drug molecules using multilayer convolutional neural networks (CNNs) and MixAttention. The substructure feature extraction module employs directed message-passing neural network (D-MPNN) to extract substructures. Then, the extracted substructure information is passed through multilayer graph attention network (GAT) and self-attention graph pooling (SAGPooling) layer to generate feature vectors containing both substructure and topology information. Subsequently, the prediction module predicts drug interactions based on these extracted features

The network architecture of SSF-DDI is illustrated in Fig. 1, and it consists of two main modules: a drug molecule sequence feature extraction module and a substructure feature extraction module. The sequence feature extraction module captures sequence features using a CNN. The substructure feature extraction module employs a message-passing neural network to extract substructures. The extracted substructure information is then passed through the substructure feature encoder to generate feature vectors containing both substructure and topology information. Finally, the extracted sequence feature information and substructure feature information are input to the prediction module to obtain the final prediction results.

### Drug substructure graph feature encoder: SGFE

RDKit converts SMILES to an undirected graph $G = \{ V,E\}$ [28], where *V* denotes the set of nodes and denotes the edges in the graph structure. In this context, atoms are represented as nodes, while chemical bonds are depicted as edges that connect the nodes within the graph structure. The dimensions of features used for atoms and bonds can be found in Table 1.

**Table 1:** The dimensions of features used for atoms and bonds in a molecule graph. *Source*: Adapted from [29]

Name	Dimensions	Description
Atom type	Total number of heavy atoms in the dataset	Atom type (e.g., C, O, N)
Degree	11	Count of covalent bonds
Implicit valence	7	Number of implicit H atoms attached to the atom
Hybridization	5	Hybridization rearranges electron orbitals in an atom for efficient covalent bonding (e.g.,sp, sp2, sp3)
Aromatic	1	Whether the atom is situated in an aromatic framework
Formal charge	1	Formal charge of the atom
Radical electrons	1	Number of lone electrons for the atom
Bond type	4	[single, double, triple, aromatic]
Conjugated	1	Whether the bond is involved in a conjugated arrangement
Ring	1	Whether the bond is within a closed loop structure

In the molecule, ${v_i}$ represents the *i*th atom, and ${e_{ij}}$ denotes the chemical bond between the *i*th and *j*th atoms. Each node ${v_i}$ corresponds to a feature vector ${x_i} \in {R^d}$, and each bond ${e_{ij}}$ corresponds to a feature vector ${x_{ij}} \in {R^{d'}}$. Table 2 summarises the corresponding parameters of the model components used in extracting the graph structure information of individual drugs and the transformation of the feature dimensions. Message-passing neural network (MPNN) [30] is a generalized GNN suitable for feature extraction of graph-structured data, and many recent studies have used MPNN for molecular property prediction and drug feature extraction [20, 29]. SSF-DDI uses an MPNN variant called a directed message-passing neural network (D-MPNN) [31]. D-MPNN minimizes unnecessary circular message passing by propagating messages through directed edges instead of nodes. Similar to GNNs, D-MPNN includes message-passing and readout phases.

Notice that in DMPNN, while the original graph data structure is undirected, information is passed from one node to its neighboring nodes by splitting undirected edges into two directed edges during the data preparation process. Through this approach, the model can capture interactions and relationships between nodes.

**Fig. 2:**
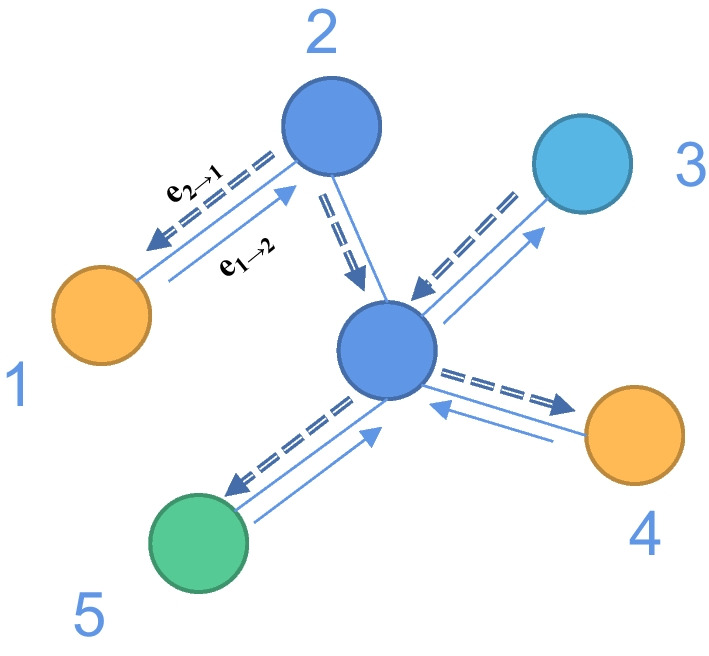
D-MPNN propagates messages through edges

D-MPNN differs from GNN in delivering messages through nodes but propagating messages through directed edges. As shown in Fig. 2, ${e_{ij}}$ represents the edge from node i to node j, ${e_{ij}}$ is different from ${e_{ji}}$ and the edge-level hidden feature is $h_i^{\left( t \right) }$. $h_{ij}$ and $message_{ij}$ refer to the bond-level features along ${e_{ij}}$. The ${t^{th}}$ iteration aggregated message delivery vector $message_{ij}^{t + 1}$ is calculated as follows:

1 $$\begin{aligned} message_{ij}^{\left( {t + 1} \right) }= & {} \mathop \sum \limits _{{v_k} \in \left( {{v_i}} \right) \backslash {v_j}} {M_t}\left( {{v_k},{v_i},h_{ki}^{\left( t \right) }} \right) , \end{aligned}$$

2 $$\begin{aligned} h_{ij}^{\left( {t + 1} \right) }= & {} {U_t}\left( {h_{ij}^{\left( t \right) },m_{ij}^{\left( {t + 1} \right) }} \right) . \end{aligned}$$

However, $m_{ij}^{\left( {t + 1} \right) }$ in Eq. 2 is independent of its reversed message $m_{ji}^t$, resulting in a more effective message passing than MPNN. The functions ${M_t}$ and ${U_t}$ are expressed as follows:

3 $$\begin{aligned} {M_t}\left( {{v_i},{v_i},h_{ij}^{\left( t \right) }} \right)= & {} h_{ij}^{\left( t \right) }, \end{aligned}$$

4 $$\begin{aligned} {U_t}\left( {h_{ij}^{\left( t \right) },m_{ij}^{\left( {t + 1} \right) }} \right)= & {} h_{ij}^{\left( 0 \right) } + m_{ij}^{t + 1}. \end{aligned}$$

The edge-level hidden features are initialized as follows:

5 $$\begin{aligned} {h_{ij}^{\left( 0 \right) } = {W_i}{x_i} + {W_j}{x_j} + {W_{ij}}{x_{ij}}}, \end{aligned}$$

where ${W_i} \in {R^{h \times d}},{W_j} \in {R^{h \times d}}$ and ${W_{ij}} \in {R^{h \times d'}}$ are learnable weight matrixes, ${x_i}$ and ${x_j}$ are the feature vectors of node ${v_i}$ and node ${v_j}$, respectively, and $x_{ij}$ is the feature vector of bond ${e_{ij}}$. The edge-level features are aggregated through summation during the update of the edge-level hidden features for the $i^{th}$ iteration. After $T^{th}$, the bond-level features are summed and aggregated into node-level hidden features, which are calculated as follows:

6 $$\begin{aligned} {h_i^{\left( T \right) } = \mathop \sum \limits _{{v_j} \in N\left( {{v_i}} \right) } h_{ji}^{\left( T \right) }}, \end{aligned}$$

where $N(v_i)$ represents the set of neighbor nodes of ${v_i}$, and $h_{ji}$ is based on the bond-level features of neighbor node ${v_j}$ pointing to node ${v_i}$. The detailed output dimensions and the parameters of the D-MPNN function are shown in Table 2 for the DMPNN data.

Finally, the global representation of the drug is input to a two-layer graph attention network (GAT), and its specific output size and parameters for the GATConv are recorded in Table 2. For each atom in the molecule, the similarity coefficient between it and its neighboring atoms is calculated step by step using Eq. 7.

7 $$\begin{aligned} {{e_{ij}} = Leaky{\mathop {text{Re}}\limits } LU\left( {W{h_i}\parallel W{h_j}} \right) ,j \in {N_i}}, \end{aligned}$$

where node *j* belongs to the set of neighboring nodes ${N_i}$ of node *i*. The concatenation operation is denoted as $\parallel$, and *LeakyReLU* is the activation function. $W \in {R^{F' \times F}}\;$ is a learnable matrix. Then, in order to better allocate weights, we use the function to normalize the correlations calculated for all neighbors, as shown in the following equation:

8 $$\begin{aligned} {{\alpha _{ij}} = softmax\left( {{e_{ij}}} \right) }. \end{aligned}$$

After obtaining the weight coefficients, we calculate the new feature vector of the node ${v_i}$, according to the weighted sum using the attention mechanism as in Eq. 9 and the multi-headed attention with *K* heads as in Eq. 10. The symbol $\sigma$ in Eqs. 9 and 10 is the rectified linear unit (ReLU) activation function.

9 $$\begin{aligned} h_i^{'}= & {} \sigma \left( {\mathop \sum \limits _{j \in {N_i}} {\alpha _{{\textrm{ij}}}}W{h_j}} \right) , \end{aligned}$$

10 $$\begin{aligned} h_i^{'}= & {} \mathop \parallel \limits ^{\mathop {k = 1}\limits _K } \sigma \left( {\mathop \sum \limits _{j \in {N_i}} {\alpha _{{\textrm{ij}}}}^k{W^k}{h_j}}\right) . \end{aligned}$$

Global sum pooling function is employed to obtain the graph-level representation *G*. However, meaningful information about some substructures may be overlooked in the graph-level drug molecule-based representation, making secondary substructures’ information overshadow significant substructures’ information. To address this issue, we adopt self-attention graph pooling (SAGPooling) [32] as the final readout function. Specifically, SAGPooling incorporates hierarchical pooling and self-attention mechanisms to distinguish between nodes that should be removed and retained, considering both the molecule’s atomic features and topological structure features, whose specific output size and parameters for the SAGPooling are recorded in Table 2. For each GAT layer *l*, the final readout function is as follows:

11 $$\begin{aligned} {h_d^{\left( l \right) } = sigmoid \left( {\mathop \sum \limits _{j = 1}^N {\lambda _j}h_j^{\left( l \right) }} \right) ,{\lambda } = softmax(A{X^{\left( l \right) }}W_{SAG}^{\left( l \right) })}, \end{aligned}$$

where *A* is the adjacency matrix, $X^{\left( l \right) }$ is the embeddings of all nodes in the $l^{th}$ layer’s, $W_{SAG}^{\left( l \right) } \in {R^{d(l)\times 1}}$ are $l^{th}$ layer’s trainable matrix, *d* is the input dimension of node-level features.

**Table 2:** Model structure and parameters for drug graph information extraction

Layer name	Output size	Parameters
DMPNN	Atom feature dim 64	In_features 70, out_features 64, hidden dim 64, n_iter 10
GATConv	Atom feature dim 70	In_channels 70, out_channels 35, heads 2
SAGPooling	[batch_size,64]	In_features 64,out_features 1

### Drug molecule sequence feature extraction

Table 3 summarises the corresponding parameters of the model components used in the extraction of sequence information for individual drugs and the transformation of the feature dimensions. Convolutional layers can extract representational features. These layers move a fixed-size kernel over the input to obtain spatially relevant information and introduce common features [13]. The proposed model uses CNN blocks for feature extraction on the input SMELES drug sequence. First, the drug’s feature embedding is performed by using the embedding layer. In extracting drug sequence features, the SMILES representation of drugs is mapped to embedding vectors. It is achieved by defining a mapping from characters to indices and setting a maximum length for the SMILES strings, facilitating the conversion of SMILES strings into numerical representations. The maximum SMILES length is set to 100 throughout this process, resulting in embedded vectors representing sequence information with dimensions [batch_size, 100]. Prior to serving as input to the attention module, the embedded vectors undergo an additional step where they are passed through an embedding layer to downscale the drug’s features to a specified dimension, whose specific output size and parameters for the Embedding Layer are recorded in Table 3. In this study, we achieved the best results with a dimension of 64. Then, the CNN module is used to realize the sequence feature extraction of the drug, as shown in Fig. 1 and Eq. 12, where ${X^i}$ denotes the input of the *ith* convolutional layer, ${W^i}$ denotes the parameters of the *ith* convolutional kernel, and ${Z^i}$ denotes the output of the *ith* convolutional layer. Three 1D convolutions are used for feature extraction of the sequence, and each convolutional layer is followed using the ReLU activation function to improve the nonlinear capability of the model.

12 $$\begin{aligned} {Z^i} = ReLU(Conv1d({X^i},{W^i})). \end{aligned}$$

The input dimension of the first convolutional layer is 64, with an output dimension of 40 and a convolutional kernel size of 4. The second convolutional layer has an input dimension of 40, an output dimension of 80, and a convolutional kernel size of 6. The input size of the third convolutional layer is 80, with an output dimension of 160 and a convolutional kernel size of 8. After passing through this CNN, the dimensions of the features for the two drugs become [batch_size, 160, 85]. In addition, the specific output about the convolution of each layer and the parameters are shown in Table 3 for the data of Conv1, Conv2 and Conv3. Then, we extract the key features of the sequence information using the Mix Attention module. MixAttention module consists of drug-pair attention, where both drugs share the same weights. We find that the best experimental results are achieved when the head hyperparameter is 4 in our experiments, whose specific output size and parameters for the MixAttention Layer are recorded in Table 3. The attention values of different heads of drug A for drug B are computed separately for a given convolutional feature ${D_1}$ of drug A and convolutional feature ${D_2}$ of drug B. Next, we splice the computed results. The computational formulas are as follows:

13 $$\begin{aligned} MixAttention({D_1},{D_2},{D_2})= & {} Concat(hea{d_1}, \ldots ,hea{d_4}), \end{aligned}$$

14 $$\begin{aligned} hea{d_i}= & {} Attention\left( {{D_1}W_i^{{D_1}},{D_2}W_i^{{D_2}},{D_2}W_i^{{D_2}}} \right) . \end{aligned}$$

We calculated the attention value using the following formula:

15 $$\begin{aligned} Attention({D_1},{D_2},{D_2}) = softmax(\frac{{{D_1} * {D_2}^T}}{{\sqrt{d} }} * {D_2}), \end{aligned}$$

where *d* denotes the input dimension of the convolutional features. Notably, computing the MixAttention value of drug B concerning drug A is vital, enabling cross-feature interactions between drug-pair.

Finally, the obtained MixAttention values corresponding to different drugs are spliced and integrated into the final feature representation as follows:

16 $$\begin{aligned} {Y^{out}}= & {} concat\left( {{Y^1},{Y^2}} \right) , \end{aligned}$$

17 $$\begin{aligned} {Y^i}= & {} \mathrm{{maxpooling}}\left( {{Z^i} * 0.5 + MixAttentio{n^i} * 0.5} \right) , \end{aligned}$$

where maxpooling denotes executing a maximum pooling operation, obtaining the sequence feature ${Y^{out}}$ of drug-pair. The maxpooling layers downsample the sequence feature of drug-pair to generate 1D feature vectors. The dimension of the maxpooling layers is set to 85, and its specific parameters for the Maxpooling Layer are recorded in Table 3.

**Table 3:** Model structure and parameters for drug sequence information extraction

Layer name	Output size	Parameters
Embedding Layer	[batch_size, 100,64 ]	num_embeddings 65, embedding dim 64
Conv1	[batch_size,40,97]	In_channels 64, out_channels 40, kernel 4, stride 1
Conv2	[batch_size,80,92]	In_channels 40, out_channels 80, kernel 6, stride 1
Conv3	[batch_size,160,85]	In_channels 80, out_channels 160, kernel 8, stride 1
MixAttention Layer	[batch_size,160,85]	In_features 160, out_features 160
Maxpooling Layer	[batch_size,160]	Kernel_size 85, stride 85

### Drug–drug interaction prediction

We extracted the sequence and substructure information of drug-pair by Drug Substructure Graph Feature Encoder and Drug Molecule Sequence Feature Extraction module, respectively. We concatenate the extracted sequence and substructure information in the prediction module, obtaining a final feature vector. This vector is then fed into a fully connected layer for the ultimate drug relationship prediction, thereby achieving the fusion of sequence and substructure information. The feature of drug pairs $\left( {{d_{{\textrm{x}}}},{d_{{\textrm{y}}}}} \right)$ is calculated as follows:

18 $$\begin{aligned} {d_{{x_{final}}}}= & {} concat\left[ {{d_{{x_{cnn}}}},{h_{{x_d}}}} \right] , \end{aligned}$$

19 $$\begin{aligned} {d_{{y_{final}}}}= & {} concat\left[ {{d_{{y_{cnn}}}},{h_{{y_d}}}} \right] . \end{aligned}$$

We modeled the prediction of DDI as a binary classification task. Given a DDI tuple $\left( {{d_{{\textrm{x}}}},{d_{{\textrm{y}}}},r} \right)$, the likelihood of DDI prediction was calculated as follows:

20 $$\begin{aligned} P\left( {{d_{{\textrm{x}}}},{d_{{\textrm{y}}}},r} \right) = \varsigma \left( {{u_r} \odot MLP\left( {Concat\left( {{d_{{x_{final}}}},{d_{{y_{final}}}}} \right) } \right) } \right) , \end{aligned}$$

where $\varsigma$ is the sigmoid activation function.

The representation of specific types of drug interactions is denoted as ${u_r} \in {R^b}$. The minimum cross-entropy loss function [33] is defined as the loss function, calculated as follows:

21 $$\begin{aligned} L = \frac{1}{{\left| \eta \right| }}\sum \limits _{\left( {{d_{{\textrm{x}}}},{d_{{\textrm{y}}}},r} \right) \in \eta } {{z_i}log({p_i}) + (1 - {z_i})log(1 - {p_i})}, \end{aligned}$$

when ${z_i} = 1$, the drug exhibits an interaction. The symbol ${p_i}$ denotes the predicted probability of DDI, $\eta$ is DDI tuples in the dataset.

## Experiments

### Dataset

We evaluated SSF-DDI’s performance in two real-world datasets: DrugBank and TWOSIDES. DrugBank contains bioinformatics, chemoinformatics, and other resources incorporating detailed drug data [34], covering 86 different interaction types and describing how one drug affects the metabolism of other drugs. It contains 1706 drugs with 191808 DDI triplets. We represented each drug as SMILES and converted it to a molecular map using RDKit. We used the data segmentation scheme from GMPNN-CS [32] for the transduction and generalization setups. The TWOSIDES dataset [35] contains 645 different drugs, 963 interaction types, and 4576287 DDI triplets. This dataset was obtained after filtering and preprocessing the raw TWOSIDES data. Unlike DrugBank, these interactions are at the phenotypic level.

### Experimental settings

In this paper, we evaluate the performances of the DDI prediction model using 3-fold cross-validation for a more robust evaluation of the method. We view the DDI prediction as a binary classification problem where each data sample contains two drugs labeled as either interacting or not interacting. In the training set, positive samples are labeled with a “1”, while samples that do not interact (negative samples) are labeled with a “0”. In our experiments, we trained and evaluated the model according to the parameter settings listed in Table 4 Six metrics are selected to evaluate the proposed model: Accuracy (ACC), Area Under the ROC Curve (AUC), F1 value (F1), precision (Precision), recall (Recall), and average accuracy (AP).

**Table 4:** Parameters of model experiments

Parameters	Value
Epoch	200
Learning rate	1e−3
Batch size	256
Weight decay	5e−4
Loss function	BCELoss
Drug embedding dimension	64
Drug length	100
Number of attention heads	5
Number of Graph Attention Convolution layers	3
Convolutional kernel size	[4,6,8]

### Performance of SSF-DDI in comparative experiments

We compared the proposed SSF-DDI with state-of-the-art methods that relied on chemical structure or sequence information as input for experiments in transductive and inductive settings. These methods comprise the following:

CNN-DDI [36] uses a CNN to realize DDI prediction based on drug sequence input.

MR-GNN [16] uses a GNN based on a multi-resolution architecture and a dual-graph state long short-term memory network to predict entity interactions.

SSI-DDI [6] is based on drug molecular substructure extraction and the calculation of substructure interactions for drug interaction prediction.

GAT-DDI [37] uses a graph attention network for DDI prediction.

GMPNN-CS [19] learns size-adaptable chemical substructures for DDI prediction via a gated information-passing neural network.

DGNN-DDI [20] uses a GNN incorporating a substructure attention mechanism for DDI prediction.

#### Performance evaluation under the transductive setting

We performed a random split of the entire dataset in the transductive setting, and every drug in the test set was likely to be found in the training set. We randomly divided the dataset into training (60%), validation (20%), and test (20%) sets. All methods share the same training, validation, and test sets.

As shown in Tables 5 and 6, the comparative experimental results show that SSF-DDI surpasses other baselines on both DrugBank and TWOSIDES under the transductive setting. The SSF-DDI exceeds DGNN-DDI by a notable margin in two datasets, which reveals the validity of the proposed method. It is worth noting that DGNN-DDI achieved the highest AUC value (98.94%) on the DrugBank dataset. However, the proposed SSF-DDI approach exhibits an AUC value very close to this figure (98.92%) while also demonstrating significant improvements across other metrics.

On the DrugBank dataset, SSF-DDI showcased remarkable superiority across multiple evaluation metrics. Compared with GMPNN-CS, which only captures graph structure information, SSF-DDI effectively solves this shortcoming by combining sequence topological and graph structure information, improving 1.14% in ACC and 1.1% in F1. In contrast to GMPNN-CS, CNN-DDI exclusively relies on sequence data for DDIs prediction. SSF-DDI can effectively mitigate the constraints stemming from the underutilization of substructure information, resulting in a significant performance boost of 1.8% in ACC and 1.69% in F1 compared to CNN-DDI.

The superiority of SSF-DDI was further validated on the TWOSIDES dataset. With an ACC of 87.30%, AUC of 93.09%, F1-score of 88.17%, Precision of 82.48%, Recall of 94.37%, and an AP of 90.47%, SSF-DDI consistently demonstrated its robustness across different datasets. Its ability to effectively combine sequence and substructure features likely contributes to its success in accurately predicting DDIs.

In conclusion, the results suggest that SSF-DDI effectively leverages both sequence and substructure features to capture nuanced drug interactions, leading to its superior predictive accuracy compared to the comparative methods. Its balanced ACC and Rec, coupled with consistently higher values across multiple metrics, position SSF-DDI as a promising improvement in the field of DDI.

**Table 5:** Comparative results of SSF-DDI on the DrugBank dataset in transductive setting (%)

Method	ACC	AUC	F1	Prec	Rec	AP
CNN-DDI	94.65	98.35	94.81	92.06	97.72	97.93
MR-GNN	93.23	97.31	93.39	91.14	95.76	96.45
SSI-DDI	92.48	97.01	92.65	90.59	94.8	96.11
GAT-DDI	92.03	96.28	92.29	89.47	95.29	94.64
GMPNN-CS	95.31	98.45	95.4	93.58	97.29	97.91
DGNN-DDI	96.09	**98**.**94**	96.16	94.72	97.88	98.51
SSF-DDI (ours)	**96**.**45**	98.92	**96**.**5**	**95**.**22**	**97**.**89**	**98**.**53**

The best results are highlighted in bold

**Table 6:** Comparative results of SSF-DDI on the TWOSIDES dataset in transductive setting (%)

Method	ACC	AUC	F1	Prec	Rec	AP
CNN-DDI	85.75	92.16	86.67	81.39	92.68	89.5
MR-GNN	85.39	91.93	86.46	80.57	93.28	89.32
SSI-DDI	82.21	89.27	83.11	79.1	87.56	86.19
GAT-DDI	67.32	75.16	63.7	71.54	57.62	72.5
GMPNN-CS	86.96	92.94	87.85	82.2	94.35	90.38
DGNN-DDI	85.29	91.92	86.12	81.51	91.28	89.41
SSF-DDI (ours)	**87**.**3**	**93**.**09**	**88**.**17**	**82**.**48**	**94**.**37**	**90**.**47**

The best results are highlighted in bold

#### Performance evaluation in the inductive setting

As depicted in Tables 5 and 7, the scores for all evaluation metrics demonstrated a significant decrease compared to the transductive setting due to the inclusion of unseen drugs in the DDI triad within the test set. These results show that predicting DDI using the inductive setting is more challenging than predicting DDI using the transductive setting. In such scenarios, evaluating the model’s generalization ability is difficult because there is no a priori knowledge of any unknown drug during training. To solve this challenge, we used the same scheme in [10] to partition the dataset. Specifically, we randomized 20% of the drugs as unknown and others as known. In the training dataset, positive and negative samples are two DDI triples in the known drugs. For the test set, we divided the positive and negative samples in the test set to contain one unknown drug and one known drug each. Thus, the task becomes to predict the DDI between a new drug and another known drug, which aligns more with real application scenarios. We adopted the approach proposed by Yang et al. [29] for two settings: the random segmentation of drugs, and the drug segmentation by structural features, which is more arduous as the drugs in the training and test sets have a significant difference in structure.

Table 7 summarizes the experimental results under the inductive setting, revealing a significant decrease in performance compared to the transduction setting. Additionally, the structure-splitting scheme is more difficult to train than the random division scheme, which is consistent with the structure splitting, preventing the drug structure information from leaking into the test set [10]. In this experiment, our methods deliver superior performance under the generalization setting. For instance, our method significantly improves various evaluation metrics compared to GMPNN-CS in experiments conducted on the dataset divided according to the structure. Specifically, the ACC improves by 5.65%, the AUC improves by 4.03%, the F1 improves by 8.13%, the Prec improves by 5.87%, the Rec improves by 8.25%, and the AP improves by 5.33%. It indicates that extracting only key substructure information cannot improve the model’s drug interaction prediction performance. In this case, additional fusion of information from other sources is required. Notice that, in a transductive experiment, the drugs in the test set have not been encountered during the model’s training phase. Owing to the presence of unknown drugs in the test set, the model’s performance on these drugs might be suboptimal, consequently affecting the Recall metric. This scenario can also result in an increased number of false negatives, thereby reducing the Rec metric. These results highlight the effectiveness of the SSF-DDI structural feature encoder in accurately capturing the structural characteristics of drug molecules, demonstrating the practical value and utility of our approach in real-world applications of DDI prediction.

**Table 7:** Comparative evaluation in inductive setting (%)

Setting	Method	ACC	AUC	F1	Prec	Rec	AP
Random Split	CNN-DDI	70.64	82.95	61.61	89.1	47.11	83.79
	MR-GNN	75.99	84.85	72.3	85.52	62.68	84.89
	SSI-DDI	75.13	83.26	72.36	81.52	65.15	83.48
	GAT-DDI	77.94	86.58	75.28	85.63	67.16	85.81
	GMPNN-CS	79.95	89.34	77.22	89.33	68.02	89.25
	DGNN-DDI	77.07	87.35	73.03	88.08	62.07	86.97
	SSF-DDI(ours)	**81**.**93**	**92**.**98**	**78**.**88**	**94**.**89**	**67**.**5**	**93**.**38**
Structure-based Split	CNN-DDI	64.12	72.87	50.52	81.91	36.24	73.65
	MR-GNN	67.33	76.52	59.71	78.41	48.59	75.25
	SSI-DDI	68.52	77.41	62.06	78.63	51.43	77.14
	GAT-DDI	71.55	80.71	65.91	82.23	55.02	80.44
	GMPNN-CS	71.57	81.9	63.83	87.68	50.21	82.9
	DGNN-DDI	70.31	85.11	59.41	93.86	43.45	86.71
	SSF-DDI(ours)	**77**.**22**	**85**.**93**	**71**.**96**	**93**.**55**	**58**.**46**	**88**.**23**

The best results are highlighted in bold

### Ablation experiment

We conducted a comprehensive ablation study to investigate the importance of each SSF-DDI component and the impact of each component of SSF-DDI on the overall performance. We conducted ablation experiments by training SSF-DDI without specific components and comparing it to the full model.

First, we evaluated the SSF-DDI and three variants of the SSF-DDI model on the DrugBank dataset, in which model variables were summarized as follows:

SSF-DDI w/o substructure removes structural features and uses sequence features.

SSF-DDI w/o sequence removes sequence features and uses SGFE for molecular structure feature extraction.

SSF-DDI w/o SGFE and sequence removes sequence features and uses D-MPNN instead of SGFE to extract molecular structure features.

**Table 8:** Comparative results of SSF-DDI (sequence and substructure features for drug–drug interactions prediction) on the DrugBank dataset (%)

Method	ACC	AUC	F1	Prec	Rec	AP
SF-DDI w/o substructure	94.77	98.34	94.92	92.23	97.76	97.83
SF-DDI w/o sequence	96.09	98.78	96.15	94.74	97.59	98.29
SSF-DDI w/o SGFE and sequence	95.56	98.58	95.63	94.05	97.27	98.15
SSF-DDI(ours)	**96**.**45**	**98**.**92**	**96**.**5**	**95**.**22**	**97**.**89**	**98**.**53**

The best results are highlighted in bold

As presented in Table 8, the comprehensive architecture of SSF-DDI demonstrates superior performance compared to all other variants, achieving ACC, AUC, and F1 score indicators of 96.45%, 98.92%, and 96.50%, respectively. These values are 1.68%, 0.58%, and 1.58%, respectively, higher than those obtained by the model solely features by 0.36%, 0.14%, and 0.35%, respectively. These findings validate that integrating drug sequence features with drug molecular graph structural features enhances the DDI prediction performance.

Table 8 also presents the model’s ACC, AUC, and F1 scores using the SGFE module, which achieves impressive values of 96.09%, 98.78%, and 96.15%, respectively. Compared to the model using MPNN without SGFE, where equal importance is assigned to each substructure, the impact of significant substructures on the prediction performance is not highlighted. The SGFE module effectively addresses this issue by incorporating an attention mechanism to enhance the representation of crucial substructures. As a result, the ACC increased by 0.53%, the AUC by 0.2%, and the F1 score by 0.52%. These improvements provide substantial evidence of the effectiveness of our designed SGFE module in enhancing the model’s prediction performance.

### Case study

To improve the credibility of the data evidence, we conducted additional case studies for drugs not available in the original dataset. Specifically, we selected several new samples from the Drugbank dataset in August 2022 for verification and augmentation. These new samples were absent from the training, validation, and test sets employed in our previous experiments. We tested these positive samples using our SSF-DDI model, trained on the original dataset. Our model successfully predicted multiple samples, as evidenced by the findings.For instance, Benzodiazepine (DB12537) are widely used and more effective than placebos in treating anxiety symptoms and improving sleep latency [38]. We selected the top 10 drugs from DrugBank with which it has drug–drug interactions, as shown in Table 9 specifically, drug interactions such as “increased risk or side effects when alfentanil is used in combination with 1,2-benzodiazepines”, “increased risk or severity of central nervous system depression when alemtuzin is used in combination with 1,2-benzodiazepines”, and so on. Notably, Benzodiazepine has not been learned in our database, and our model accurately predicted the drug interactions in Table 9, and the predictions are consistent with those shown in DrugBank. This validation study provides compelling evidence that our model has the potential to identify DDI in novel scenarios, further establishing the robustness and efficacy of our approach.

**Table 9:** Top 10 Drug Interactions for Benzodiazepine (DB12537) in DrugBank

Drugbank ID	Drug name
DB11932	Abametapir
DB00819	Acetazolamide
DB01063	Acetophenazine
DB06594	Agomelatine
DB00802	Alfentanil
DB01246	Alimemazine
DB00918	Almotriptan
DB00969	Alosetron
DB00404	Alprazolam
DB01616	Alverine

## Discussion

In this study, we have demonstrated the superiority of SSF-DDI in DDI prediction. We introduced SGFE, an effective feature extraction method capable of capturing molecular and structural features of drug atoms and important substructures. In the context of DDI prediction, we integrated drug sequence information and drug molecular graph structural information, enabling the capture of a broader range of feature information for enhanced DDI prediction accuracy. The case studies demonstrate that SSF-DDI can recognize drug–drug interactions (DDIs) in novel scenarios beyond the dataset. Moreover, SSF-DDI is capable of identifying significant substructure information related to DDIs. The accurate prediction of drug interactions has significant implications in multiple domains and holds considerable application value. This predictive capability can support physicians in making safer treatment decisions in clinical settings. Moreover, in the drug discovery field, such predictions can accelerate the drug development process and reduce medical research and development costs by enabling the anticipation of unforeseen interactions resulting from the introduction of novel drugs.

**Fig. 3:**
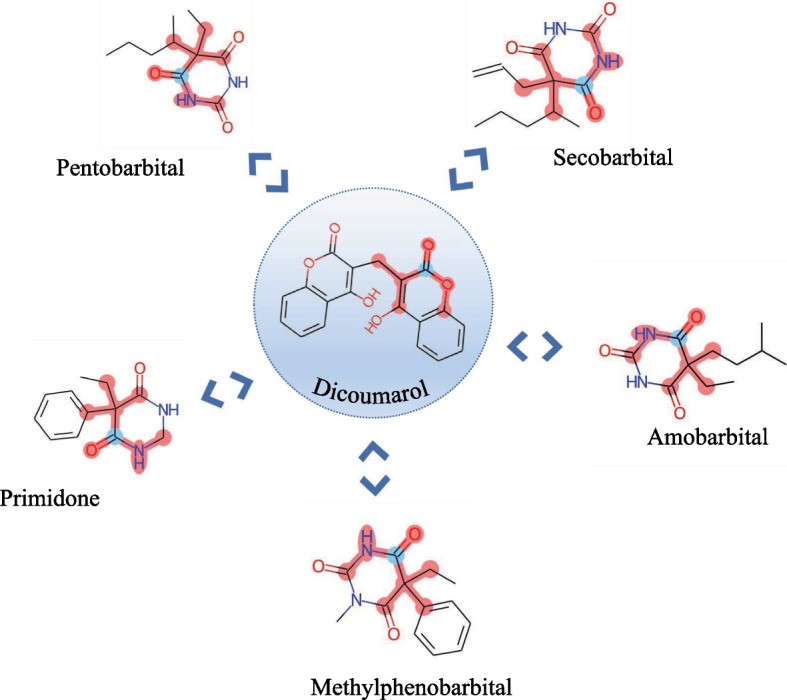
Visualization of the essential substructures of bicoumarin and the interactions with five other drugs. Atomic centers are marked in blue and corresponding substructures are marked in red

To further investigate the acquired drug structure information and determine the key components influencing DDI prediction, we employed the SAGPooling operation to obtain the contribution scores of individual atoms in the drug molecules. By selecting the substructures associated with the highest attention scores, we visualized the significant substructures of bicoumarin compared to the other five drugs, as depicted in Fig. 3. Notably, SSF-DDI successfully identified the effective substructures of pentobarbital, amobarbital, secobarbital, primidone, and methylphenobarbital, which exhibited similar active substructures, namely barbituric acid. This finding concurs with previous experimental evidence that drugs containing barbituric acid substructures can enhance human liver microsomal activity, thereby reducing the efficacy of bicoumarin [39]. Consequently, these noteworthy atoms and substructures learned by the SSF-DDI approach demonstrate alignment with experimental and pharmacological findings.

Although the proposed SSF-DDI framework has proved effective in enhancing drug characterization and improving DDI prediction, SSF-DDI only considers drug characterization but ignores the relationship between drug actions in the complex physiological environment of the human body based on the influence of histological information such as cell structure, genes, and proteins. In future research, we will focus on integrating multi-omics information to better model DDI in the human body.

## Conclusion

In this paper, we propose a deep learning-based model for DDI prediction, called SSF-DDI, to address the problem that DDI prediction methods for drug molecule structure maps mainly focus on topological information between drug molecules but fail to utilize features of drug molecule sequences. Meanwhile, for the extraction of structural features of drug maps, we design a new feature encoder for drug substructure features. In the experiments, SSF-DDI outperformed the current state-of-the-art methods on the given datasets in two different scenarios. A case study on predicting drugs absent from the original dataset demonstrates that our method yields accurate predictions, indicating its potential to identify DDI in novel scenarios. Furthermore, visual experiments based on important atomic substructures demonstrated the ability of our method to capture crucial substructure information, thereby enhancing the interpretability of our approach. SSF-DDI is a predictive model that accurately predicts DDIs and possesses the potential to drive drug development efforts.

## Data Availability

The data used in this study were obtained from the DrugBank dataset (https://go.drugbank.com/), the TWOSIDES dataset (http://snap.stanford.edu/decagon).

## Code availability

The source code is available at GitHub (https://github.com/ZHJING25/SSF-DDI).
